# Mental Health Smartphone Apps: Review and Evidence-Based Recommendations for Future Developments

**DOI:** 10.2196/mental.4984

**Published:** 2016-03-01

**Authors:** David Bakker, Nikolaos Kazantzis, Debra Rickwood, Nikki Rickard

**Affiliations:** ^1^ School of Psychology and Monash Institute of Cognitive and Clinical Neurosciences Faculty of Medicine, Nursing and Health Sciences Monash University Clayton Australia; ^2^ Cognitive Behaviour Therapy Research Unit, School of Psychological Sciences Faculty of Medicine, Nursing and Health Sciences Monash University Clayton Australia; ^3^ Psychology Department Faculty of Health University of Canberra Canberra Australia; ^4^ Centre for Positive Psychology Melbourne Graduate School of Education University of Melbourne Parkville Australia

**Keywords:** mobile phones, mental health, smartphones, apps, mobile apps, depression, anxiety, cognitive behavior therapy, cognitive behavioral therapy, clinical psychology

## Abstract

**Background:**

The number of mental health apps (MHapps) developed and now available to smartphone users has increased in recent years. MHapps and other technology-based solutions have the potential to play an important part in the future of mental health care; however, there is no single guide for the development of evidence-based MHapps. Many currently available MHapps lack features that would greatly improve their functionality, or include features that are not optimized. Furthermore, MHapp developers rarely conduct or publish trial-based experimental validation of their apps. Indeed, a previous systematic review revealed a complete lack of trial-based evidence for many of the hundreds of MHapps available.

**Objective:**

To guide future MHapp development, a set of clear, practical, evidence-based recommendations is presented for MHapp developers to create better, more rigorous apps.

**Methods:**

A literature review was conducted, scrutinizing research across diverse fields, including mental health interventions, preventative health, mobile health, and mobile app design.

**Results:**

Sixteen recommendations were formulated. Evidence for each recommendation is discussed, and guidance on how these recommendations might be integrated into the overall design of an MHapp is offered. Each recommendation is rated on the basis of the strength of associated evidence. It is important to design an MHapp using a behavioral plan and interactive framework that encourages the user to engage with the app; thus, it may not be possible to incorporate all 16 recommendations into a single MHapp.

**Conclusions:**

Randomized controlled trials are required to validate future MHapps and the principles upon which they are designed, and to further investigate the recommendations presented in this review. Effective MHapps are required to help prevent mental health problems and to ease the burden on health systems.

## Introduction

A smartphone is an advanced mobile phone that functions as a handheld computer capable of running software apps. Within the last decade, smartphones have been integrated into the personal, social, and occupational routines of a substantial proportion of the global population. Over half of the population in the United States owns a smartphone and 83% of these users do not leave their homes without it [1]. Average users check their phones as often as 150 times a day [2], which reflects how smartphone apps can generate, reward, and maintain strong habits involving their use [3,4]. Apps are also capable of implementing behavior change interventions [5], which may improve users’ physical health [6], such as through promotion of physical exercise [7].

Over recent years, numerous mental health apps (MHapps) have been developed and made available to smartphone users. These apps aim to improve mental health and well-being, ranging from guiding mental illness recovery to encouraging beneficial habits that improve emotional health [8]. The demand for MHapps is strong, as evidenced by a recent public survey that found that 76% of 525 respondents would be interested in using their mobile phone for self-management and self-monitoring of mental health if the service were free [9].

MHapps and other technology-based solutions have the potential to play an important part in the future of mental health care [10], making mental health support more accessible and reducing barriers to help seeking [11]. Innovative solutions to self-management of mental health issues are particularly valuable, given that only a small fraction of people suffering from mood or anxiety problems seek professional help [12]. Even when people are aware of their problems and are open to seeking help, support is not always easily accessible, geographically, financially, or socially [13].

Smartphones are not constrained by geography and are usually used privately by one individual. This means that smartphone apps can be extremely flexible and attractive to users, empowered by the confidentiality of their engagement. Seeking help by downloading and using an MHapp is well suited to the needs of young adults and other users with a high need for autonomy [14]. Users also prefer self-help support materials if they are delivered via a familiar medium [15], such as a personal smartphone. Smartphones apps are almost always accessible to users, so they can be used in any context and in almost any environment [16]. Using these apps, users can remind themselves throughout the day of ongoing goals and motivations, and be rewarded when they achieve goals [17].

However, many MHapps have not capitalized on the strengths and capabilities of smartphones. Design principles that have led to the huge success of many physical health and social networking apps have not been utilized in the MHapp field. Furthermore, evidence-based guidelines that have been developed for other self-help mental health interventions have not been applied to many MHapps. For example, many available MHapps target specific disorders and label their users with a diagnosis. Much research has suggested that this labeling process can be harmful and stigmatizing [18].

There also appears to be a lack of appreciation for experimental validation among MHapp developers. Donker et al [8] revealed that there is a complete lack of experimental evidence for many of the hundreds of MHapps available. Their systematic review identified only 5 apps that had supporting evidence from randomized controlled trials (RCTs). A search of the Apple and Google app stores as of January 2014 reveals that none of these RCT-supported apps is currently available to consumers.

For a mental health intervention to be effective, there must be a process of rigorous experimental testing to guide development [19]. Appropriate theories of engagement and implementation should also be consulted when introducing an evidence-based intervention to the public [20]. However, such research is currently lacking. A series of recommended principles based on evidence and substantiated theories would be valuable in guiding the development of future MHapps and future RCTs. A review of the literature highlights the numerous ways by which the design, validation, and overall efficacy of MHapps could be improved.

## Methods

This review aims to provide a set of clear, sound, and practical recommendations that MHapp developers can follow to create better, more rigorous apps. As such, this review covers work from a number of different research fields, including mental health interventions, preventative health, mobile health, and mobile app design. A review of currently available MHapps was also necessary to gain a clearer idea of where improvements can be made.

Databases such as PsycInfo, Scopus, and ProQuest were consulted for peer-reviewed sources. Search terms included (but were not restricted to) “mhealth,” “anxiety,” “depression," “help seeking,” “self-help,” “self-guided,” “smartphones,” and “gamification.” Articles published between March 1975 and March 2015 were considered for inclusion. Meta-analyses and systematic reviews were sought for each relevant area of investigation. Several synoptic texts were also consulted to guide foundational understanding of theoretical concepts relating to mobile apps and product design [3,5]. Sources were excluded from the review if they did not relate directly to mental health or computerized health interventions. Because this was not a systematic review, and as such was not based on a single search of the literature, the specific number of articles found and excluded was not tracked. Furthermore, multiple searches were used to explore the concepts and formulate the recommendations presented. The lead author (DB) conducted these searches and formulated the basic recommendations. The secondary authors provided individual feedback on the review, suggested sources, and guided further searches that the lead author undertook.

Most research into mobile health has focused on validating single entrepreneurial apps, rather than pursuing rigorous RCTs to validate principles that can guide development of future apps [21]. Because of the infancy of the field, the recommendations presented in the results of this review have not been rigorously validated by RCTs in an MHapp setting. Instead, each recommendation should be treated as a guide for both development of MHapps and future research. Each recommendation could well be the target of a future RCT.

### Currently Available Apps

The recommendations explored in this review should be considered in the context of the existing range of MHapps available. The suggested recommendations are as follows: (1) cognitive behavioural therapy based; (2) address both anxiety and low mood; (3) designed for use by nonclinical populations; (4) automated tailoring; (5) reporting of thoughts, feelings, or behaviors; (6) recommend activities; (7) mental health information; (8) real-time engagement; (9) activities explicitly linked to specific reported mood problems; (10) encourage nontechnology-based activities; (11) gamification and intrinsic motivation to engage; (12) log of past app use; (13) reminders to engage; (14) simple and intuitive interface and interactions; (15) links to crisis support services; (16) experimental trials to establish efficacy. This is a recommended direction for future research. To demonstrate the necessity of such a future review or some form of accreditation system to ensure the quality of health care apps [22], the lead author conducted a brief overview of the range of currently available MHapps via a series of preliminary searches of the iTunes App Store. The search terms used included “anxiety,” “depression,” “low mood,” “mental health,” “therapy,” “relaxation,” and “self-help.” Inspection and use of the apps found in these searches revealed some major gaps in their capabilities when compared with the recommendations of this review. Table 1 compares a selection of these apps across the recommended features discussed in this review.

## Results

The recommendations formulated by this review of the literature are summarized in the following section. Recommendations 1-7 have been chiefly extrapolated from the mental health literature, and Recommendations 8-14 have origins in research on user engagement and designing apps for behavior change. Recommendations 15 and 16 are recommendations specifically related to MHapps.

It may not be possible to build every single listed recommendation into a single app. Rather, this list has been compiled based on the available evidence to guide decisions when embarking on an MHapp development project. Many currently available MHapps lack features that would greatly improve their functionality, or include features that are not optimized. Thus, the purpose of this review is to collate a list of easily followed recommendations to be used by developers when creating future MHapps.

Some of these recommendations will be relevant to informing both the interface design and the marketing of MHapps. It is important to note that the marketing of an app is tied to the way that users will interact with it [23], in the same way that pretherapy expectations can influence engagement motivation and hopefulness [24]. For example, if a user downloads an app because its description on the app store lists “relaxation,” the user will plan to use the app for relaxation purposes. When app design is mentioned in the recommendations, this is inclusive of an app’s marketing.

### Recommendations

#### Cognitive Behavioral Therapy Based

Cognitive behavioral therapy (CBT) is a type of collaborative, individualized, psychological treatment that is recognized as the most supported approach to generate behavioral, cognitive, and emotional adaption to a wide range of common psychological problems [25]. The efficacy of CBT has been supported by a comprehensive review of 106 meta-analyses across different clinical groups [26]. Other meta-analyses have found strong support for CBT as an effective treatment for a huge range of psychological disorders, including depression [27,28], generalized anxiety disorder [29], social anxiety [30], health anxiety [31], panic disorder [32], posttraumatic stress disorder [33], obsessive-compulsive disorder [34], phobias, and anxiety disorders overall [35]. Meta-analytic evidence for CBT also extends to anger expression problems [36], insomnia [37], pathological gambling [38], hoarding disorder [39], irritable bowel syndrome [40], psychosis prevention [41], and occupational stress [42].

Although CBT’s most researched application is as a therapeutic technique delivered collaboratively by a trained clinician, its principles have also been used as the foundation of many self-help support measures. Using technology is a cost-effective way to enhance the efficiency of CBT treatment [43,44], and research has already demonstrated that CBT-based self-administered computerized interventions are successful for improving depression and anxiety symptomatology in adults. A meta-analysis of 49 RCTs revealed a significant medium effect size (*g*=0.77, 95% CI 0.59-0.95) for computerized CBT (CCBT) for depression and anxiety [45]. Another meta-analysis of 22 RCTs found an even greater effect size (*g*=0.88, 95% CI 0.76-0.99) [46]. Similar findings for CCBT’s efficacy have emerged from meta-analyses that have focused on anxiety [47], depression [48], and its use with young people [49]. CCBT interventions can be administered by a mobile device and still retain their therapeutic validity [50]. RCTs have established the efficacy of CBT-based interventions delivered via smartphone apps that reduce depression [50], chronic pain [51], and social anxiety disorder [52]. CBT-based features can also be appealing to users. In an analysis of features used on a smartphone app for smoking cessation, 8 of the top 10 used features were CBT based [53], such as progress tracking and journaling (see the “Reporting of Thoughts, Feelings, or Behaviors” section).

**Table 1:** Currently available iOS apps compared across recommended features.

App	Recommended feature^a^															
	1	2	3	4	5	6	7	8	9	10	11	12	13	14	15	16
AnxietyCoach	✔	✘	✘	✔^b^	✔	✔	✔	✔	✔	✔	✘	✔	✘	✔	✘	✘
Behavioral Experiments	✔	✔	✘	✘	✔	✘	✘	✔	✘	✘	✘	✔	✘	✘	✘	✘
Breathe	✘	✘	✔	✘	✘	✘	✘	✔	✘	✘	✘	✘	✔^c^	✔	✔	✘
DBT Diary Card and Skills Coach	✘	✘	✘	✘	✔	✔	✔	✔	✘	✔	✔	✔	✔^c^	✘	✘	✘
Depression Prevention	✘	✘	✘	✘	✘	✔	✘	✘	✘	✔	✘	✘	✘	✔	✘	✘
Happify	✘	✔	✔	✔^b^	✘	✔	✔	✘	✘	✘	✔	✔	✔	✘	✘	✘
HealthyHabits	✘	✔	✔	✘	✘	✔	✘	✘	✘	✔	✔	✔	✔	✘	✘	✘
HealthyMinds	✔	✔	✔	✘	✔	✔	✔	✔	✔	✔	✘	✔	✔^c^	✔	✔	✘
HIAF	✘	✘	✔	✘	✔	✘	✔	✘	✘	✘	✘	✔	✔	✘	✔	✘
iCouch CBT	✔	✔	✘	✘	✔	✘	✘	✘	✘	✘	✘	✔	✘	✘	✘	✘
iCounselor^f^	✔	✘	✘	✘	✔	✔	✘	✔	✔^d^	✔	✘	✘	✘	✔	✘	✘
iMoodJournal	✘	✘	✔	✘	✔	✘	✘	✘	✘	✘	✘	✔	✔	✔	✘	✘
In Hand	✘	✔	✔	✘	✔	✔	✘	✔	✘	✘	✘	✘	✘	✔	✔	✘
MindShift	✔	✘	✘	✘	✔	✔	✔	✔	✔	✔	✘	✘	✘	✘	✘	✘
MoodKit	✔	✔	✘	✘	✔	✔	✘	✔	✔	✔	✘	✔	✔^c^	✘	✘	✘
Moodlytics	✘	✘	✔	✘	✔	✘	✘	✘	✘	✘	✘	✔	✔^c^	✘	✘	✘
Moody Me	✘	✘	✔	✘	✔	✘	✔^e^	✘	✘	✘	✘	✔	✔^c^	✔	✘	✘
Pacifica	✔	✘	✔	✘	✔	✔	✘	✔	✔	✔	✘	✔	✘	✔	✘	✘
Pocket CBT	✔	✔	✘	✘	✔	✘	✘	✘	✘	✘	✘	✔	✘	✘	✘	✘
SAM	✔	✘	✘	✘	✔	✔	✔	✔	✔	✔	✘	✔	✘	✔	✘	✘
Smiling Mind	✔	✔	✔	✔	✔	✘	✘	✘	✘	✘	✔	✔	✘	✔	✔	✘
Stress & Anxiety Companion	✔	✘	✔	✘	✔	✔	✔	✔	✘	✘	✘	✔	✘	✔	✘	✘
SuperBetter	✘	✔	✔	✔^b^	✘	✔	✘	✘	✔	✔	✔	✔	✔	✘	✘	✘
ThinkHappy	✘	✔	✔	✘	✘	✘	✔	✘	✘	✘	✘	✘	✘	✘	✘	✘
What’s Up?	✔	✔	✔	✘	✘	✔	✔	✔	✘	✔	✘	✘	✘	✔	✘	✘
WorkOut	✔	✔	✔	✘	✔	✔	✘	✘	✔	✔	✘		✔^c^	✔	✘	✘
WorryTime	✔	✘	✔	✘	✘	✘	✘	✔	✘	✘	✘	✘	✔	✔	✔	✘

^a^See the “Currently Available Apps” section for the 16 recommendations.

^b^Not using automated processes.

^c^Default is for reminders to be off.

^d^Only because there are separate apps for separate problems, so each app recommends activities for that target problem.

^e^Accessible via forums

^f^Includes separate iCounselor: Depression; iCounselor: Anger; and iCounselor: Anxiety apps.

Although primarily applied in clinical contexts, CBT is also fundamentally a prevention technique acting to prevent psychological problems from precipitating or maintaining clinical disorders [54-56]. This means that CBT-based MHapps have the potential to be effective for managing both clinical and subclinical psychological problems [57], provided that such apps avoid using CBT-based techniques that are used for very specific clinical psychological problems, are marketed correctly, and employ well-designed interfaces.

**Figure 1:**
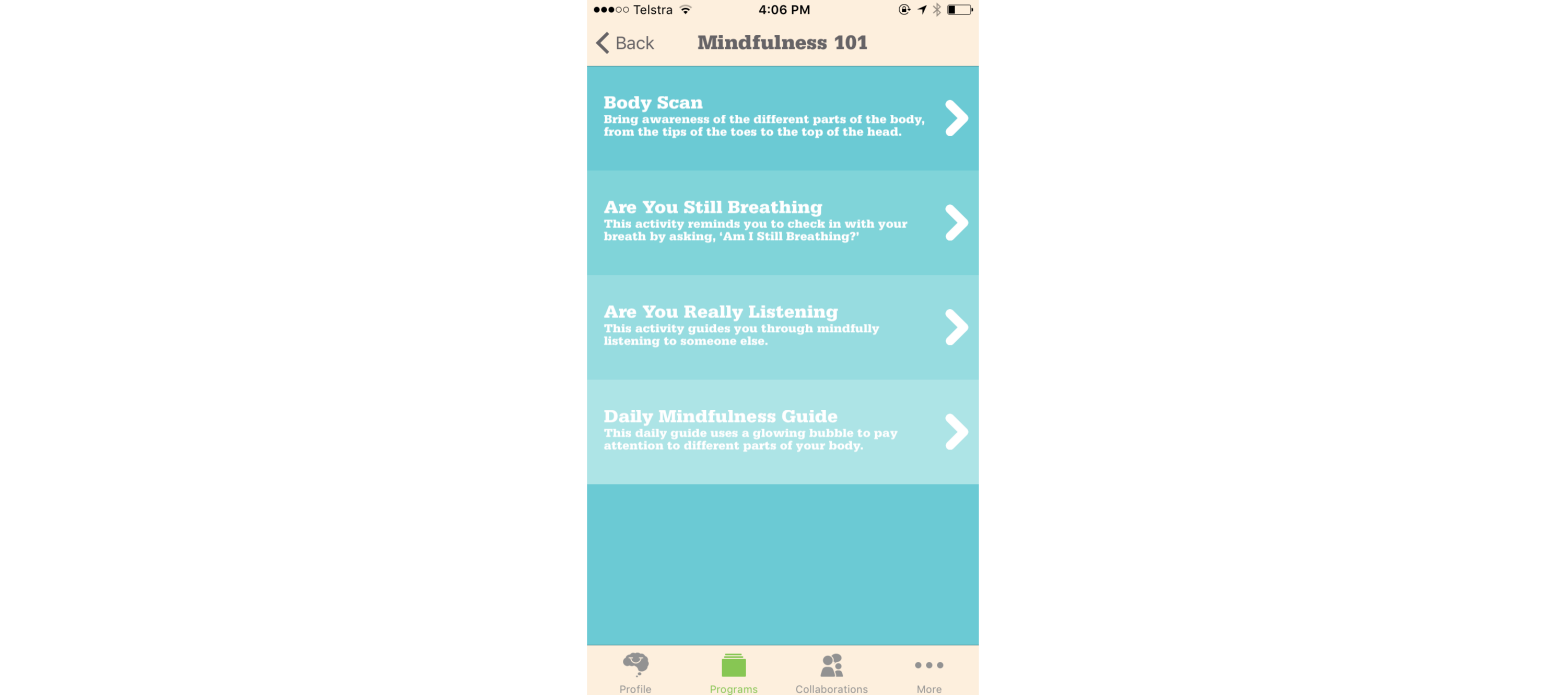
Screenshot of Smiling Mind displaying meditations.

**Figure 2:**
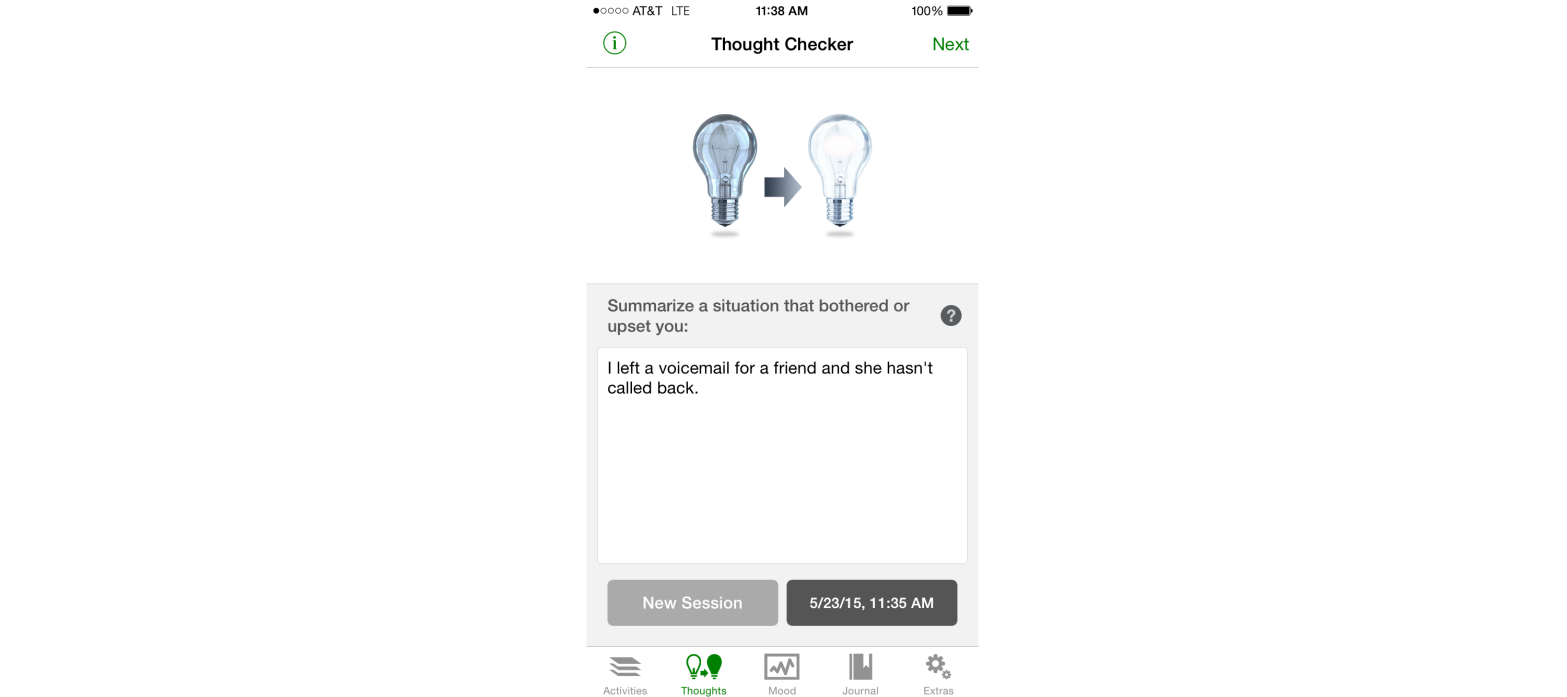
Screenshot of MoodKit displaying thought checker.

To ensure that an MHapp is indeed CBT based, it is important to keep the core principles of CBT in mind. Mennin et al [58] summarize the unifying factors that underlie all CBT approaches into three change principles: context engagement, attention change, and cognitive change. Context engagement involves training clients in a way that promotes more adaptive associative learning, which involves having them learn cues for threats and rewards that are more reasonable and lead to better functioning than existing cues. This includes CBT techniques that aim to recondition maladaptive associations, such as exposure and behavioral activation. The app SuperBetter [59] prescribes “power-ups” that may incorporate these techniques. Attention change is the ability to focus attention adaptively on relevant, nondistressing stimuli. This includes therapeutic processes such as attention training, acceptance or tolerance training, and mindfulness. These techniques are employed in Smiling Mind [60], and can be seen in the meditations displayed in Figure 1. Finally, cognitive change is the ability to change one’s perspective on an event, which then affects the emotional significance and meaning of that event [61]. This includes metacognitive awareness and cognitive distancing, which are promoted through therapeutic processes such as decentering or defusion and cognitive reframing or reappraisal. An example of this can be found in using the Thoughts tool in MoodKit [62], as seen in Figure 2. If these three change principles are being employed to some degree by an intervention, then it can claim to be based on CBT’s core principles.

To employ these change principles effectively, a therapist and client must develop a relationship that involves collaborative empiricism (CE) [63]. CE refers to shared work between client and practitioner to embed a hypothesis testing approach into interventions [64]. CE empowers clients to explore their behaviors and beliefs outside of therapy sessions using between-session (homework) interventions [65]. A meta-analysis of studies that compared therapy with and without homework found an effect size of *d*=0.48 in favor of using between-session activities [66]. In the context of CBT-based MHapps, CE may refer to how the app interacts with the user to complete therapeutic tasks, and whether it does it in a collaborative, experimentation-based way. This would ideally involve encouraging users to develop their own hypotheses about what may happen as a result of using the app or participating in certain activities (see the “Recommend Activities” section). An app that embraces CE is Behavioral Experiments-CBT [67], which affords users the ability to predict the outcomes of any behavioral experiments they participate in. Behavioral experiments are CBT-based challenges that individuals perform to challenge their own beliefs about the negative outcomes of various situations [68]. This process of comparing predictions with actual outcomes can challenge unhelpful beliefs [69].

Self-determination theory (SDT) can aid in understanding CE’s benefits in CBT [64]. SDT emphasizes the effects of autonomy and mastery on intrinsic motivation [70]. Intrinsic motivation is the “prototypic manifestation of the human tendency toward learning and creativity” [71]. Autonomy feeds this motivation by affording individuals opportunities for self-direction and choice [72], and fostering self-efficacy [73]. Self-efficacy and a feeling of competency lead to a feeling of mastery, which is an intrinsic reward and motivator in itself [74]. CE and between-session activities promote autonomy and provide opportunities for development of competence in behavioral, emotional, or cognitive self-management. SDT can inform MHapps on how to best engage users in CBT-based interventions (ie, by intrinsically motivating them). Users will be more motivated to engage with apps and products that encourage autonomy, emphasize user choice, and allow opportunities for building mastery. For example, SuperBetter [59] employs SDT-based, game-based principles to intrinsically motivate users to engage with the app and experience the well-being-promoting effects of mastery (see the “Gamification and Intrinsic Motivation to Engage” section).

#### Address Both Anxiety and Low Mood

Emotional disorders (eg, anxiety and depression) are by far the most common psychological conditions in the community, with an estimated 20.9% of US citizens experiencing a major depressive episode and 33.7% suffering from an anxiety disorder at some point throughout their lives [75]. Emotional disorders are also the most treatable [76], but help seeking for sufferers is very low [77]. There is strong supportive evidence for CCBT as an effective therapy for reducing symptoms of the most common anxiety disorders and depression [45,46].

There is an extremely high comorbidity between anxiety and depression [78], with 85% of people diagnosed with depression problems also suffering significant anxiety and 90% of people diagnosed with anxiety disorders suffering significant depression [79]. In Australia, 25% of all general practice patients have comorbid depression and anxiety [80]; whereas in Great Britain, half of all mental illness cases are mixed anxiety and depression [81]. These two diagnoses share a few major underlying factors [82]. This raises two important considerations for MHapp self-help interventions. First, interventions designed for one disorder are likely to have some efficacy for other emotional disorders, and second, interventions that target shared underlying factors across emotional disorders will be more efficacious.

Transdiagnostic CBT (TCBT) is an effective therapeutic approach that targets the common underlying factors shared by different psychological disorders. A meta-analysis of RCTs found a large effect size (standardized mean difference = −0.79, 95% CI −1.30 to −0.27) for TCBT across different anxiety disorders [83]. Furthermore, TCBT has been found to be successful in treating depression [25]. Barlow et al’s [84] Unified Protocol (UP) is a recent TCBT treatment that focuses on monitoring and adjusting maladaptive cognitive, behavioral, and emotional reactions that underlie depression and anxiety disorders. The UP has yielded very promising results across various emotional disorders, reducing psychopathology [85] and improving psychological well-being [86]. It is important to note that TCBT protocols do not imply that all emotional disorders can be treated effectively with the exact same techniques [87]. The basic structure for treating different clinical problems may be relatively uniform, but tailoring of interventions is still essential (see the “Automated Tailoring” section), and the structure of TCBT affords flexibility. For example, the UP consists of four core modules that are designed to (1) increase present-focused emotional awareness, (2) increase cognitive flexibility, (3) aid identification and prevention of patterns of emotion avoidance and maladaptive emotion-driven behaviors, and (4) promote emotion-focused exposure [88]. This enables a prescriptive approach, whereby certain modules can be focused on more than others, depending on the needs of the client or user [88]. An Internet-delivered TCBT intervention called the Wellbeing Program used a structure of 8 lessons, focusing on areas such as psychoeducation, thought-monitoring strategies, behavioral activation, and graded exposure [57]. A clinician guided users through the program and tailored the delivery of each lesson to the user’s needs. An RCT supported the efficacy of this intervention across depression and anxiety disorders [57]. Although the Wellbeing Program was guided by a clinician and not via automated processes, many other self-guided CBT interventions use a transdiagnostic approach to maximize efficiency and adaptability [89], particularly in an automated Internet-delivered context [90].

Despite the success of TCBT, many MHapps are designed for the treatment of specific disorders. Some apps are marketed for anxiety and others for depression. Few apps acknowledge that the underlying CBT principles guiding self-help interventions for anxiety and mood problems are very similar; thus, broadening the target group of the app can be beneficial for all users. Combining treatments for both anxiety and depression into a single app would also reduce the commitment required for engagement. Users could consolidate their investment within a single app, instead of dividing their effort and time engaging with 2 separate apps (one for anxiety and the other for depression).

#### Designed for Use by Nonclinical Populations

Many apps have been designed for use with populations who have been diagnosed with a specific clinical disorder, from depression (eg, Optimism [91]) and anxiety (eg, SAM [92]) to eating disorders (eg, Recovery Record [93]) and borderline personality disorder (eg, DBT [Dialectical Behavior Therapy] Diary Card and Skills Coach [94]). Some of these clinical diagnosis apps are known to be effective for interventions [8], but they do not capitalize on one of the major advantages of smartphones: high accessibility. Smartphones are interwoven into the routines of millions of people all over the world, the majority of whom have not been diagnosed with a clinical psychological disorder but do experience unpleasant psychological distress from time to time. Targeting a specific clinical population with an MHapp automatically excludes the majority of smartphone owners from using that app. By contrast, an MHapp built for a population interested in the *prevention* of emotional mental health problems increases the number of eligible and willing users. A meta-regression of 34 studies found that self-help interventions were significantly more effective when recruitment occurred in nonclinical settings (effect size *I* ^2^=0.66) than in clinical settings (effect size *I* ^2^=0.22) [48]. The field would therefore benefit from more MHapps with preventative applications that are widely marketable, rigorous, and effective.

An MHapp market saturated with clinical diagnosis apps also has the potential to be harmful for help seekers. Users who are experiencing low-level symptoms of a disorder may feel labeled by an app that assumes that they have a clinical diagnosis [95]. Self-stigma from this labeling can be harmful, lowering self-esteem and self-efficacy [96]. Initiatives that acknowledge the continuum of mental health and the importance of well-being promotion may reduce stigma and increase help seeking for mental health problems [97]. Programs such as Opening Minds [98] aim to reduce mental illness stigma by adopting a nonjudgmental, nondiagnostic, and nonclinical CBT-based stance to mental health problems. MHapps that focus on nonclinical mental health, psychological well-being, or coping abilities may therefore avoid the harmful effects of labeling mental illness [99].

CBT is built on the foundation that mental health is a continuum [89] and that supporting individuals in coping with nonclinical psychological distress can prevent symptoms from reaching clinical significance [100]. Furthermore, CBT-based support can help prevent relapse [101], expand an individual’s coping skills repertoire [102], and assist individuals experiencing psychological distress to avoid developing a clinical disorder [103]. Building a CBT-based MHapp that acknowledges the continuum of mental health can be used by both clinical and nonclinical populations.

CBT treatment adopts a formulation-based approach rather than a diagnosis-based approach [54,104]; as such, a diagnosis is not necessary for support to be given. Formulation involves exploring the predisposing, precipitating, perpetuating, and protective factors connected to a psychological problem, and then building these factors into a causal model [105]. Conversely, diagnosis relies on detection of symptoms and fulfillment of criteria statistically linked to a particular disorder [106]. In many cases, a formal diagnostic label is not important for informing real-world treatment, and it does not specify the causal factors contributing to an individual’s unique psychological problems. Formulation is much more useful because it can inform exactly which precipitating and perpetuating factors are contributing to an individual’s unique psychological problem, and which psychological techniques can produce optimal solutions [107]. Hofmann [108] proposed a cognitive behavioral approach for classifying clinical psychological problems that avoids diagnostic labeling, which is better at informing CBT-based support because it is based on formulation. MHapp developers are encouraged to explore formulation-based approaches to CBT to inform the development of CBT-based MHapps.

Designing MHapps for nonclinical support may mean adopting a preventative framework. There are generally three types of preventative intervention: universal (ie, delivered to everyone in the community), selective (ie, delivered to at-risk groups), and indicated (ie, delivered to individuals with preclinical symptoms) [109]. The flexibility of MHapps means that a single app could theoretically adapt to any of these three intervention models, providing a universal intervention as default, and tailoring to a selective or indicated approach if a user’s responses suggest that they are at risk of a certain condition.

Some mobile interventions that have been validated and trialed experimentally were built for personal digital assistants (PDAs) and not for modern smartphones [7,110]. This severely limits their nonclinical use and introduces other barriers to routine engagement that are not experienced by smartphone apps. However, evidence and principles from PDA-based studies should be considered when designing smartphone apps.

#### Automated Tailoring

An advantage of eHealth interventions over other self-help interventions is their capacity for tailoring [90,111]. Tailoring in this context refers to the adjustment of technology-delivered self-help programs to suit the user’s needs, characteristics, and comorbidities or case formulation [112]. Tailored CCBT interventions have been shown to be more efficacious than rigid self-help interventions across a range of depressive and anxiety disorders [112-115].

Formulation-based tailoring improves the functionality of an intervention and provides targeted solutions to a user’s psychological problems. There is a large range of different self-help mental health interventions available, and selecting the right intervention can be a challenging and overwhelming process [15]. The complexity of choices can be simplified or reduced by building an app capable of automated tailoring, which combines elements of a large number of different interventions and deploys them strategically depending on the needs of individual users. A review of currently available MHapps reveals, however, that many apps aim to provide a service but do not service a need [116]. For example, many apps provide guided meditation, but do not guide users toward meditation when they are feeling anxious. With tailoring, the app can recommend users specific solutions to their specific problems.

Automated tailoring requires the collection of data to identify the needs of users and develop a functional analysis or case formulation. This can be achieved in three main ways. First, self-report measures can be deployed to elicit in-depth responses about symptoms and characteristics. Second, data from a user’s self-monitoring (see “Reporting of Thoughts, Feelings, and Behaviors” section) can be used to predict the types of interventions that are well suited to an individual user. Third, an app’s behavioral usage data can be used to predict which features of that app a user is using most. If these second and third data sources are correctly utilized, tailoring can be carried out seamlessly, without any additional input from the user, which decreases users’ required effort to use the app and thereby increases app functionality [3].

CBT includes a very wide range of evidence-based techniques that may be selectively employed by an MHapp depending on automated tailoring data. For example, if data sources suggest that the user is experiencing significant physiological arousal, rather than overwhelming worry or other anxiety-related problems, CBT techniques such as breathing relaxation may be recommended over others, based on the available evidence [117]. Ideally, these therapeutic techniques would be employed by the MHapp that actually performs the automated tailoring, but restrictions may mean that the MHapp must rely on referring users to other apps. This is not ideal, as it may disrupt the user’s engagement with the MHapp. However, if necessary, any referrals should be based on a thorough review of the other existing apps and their supporting evidence [116].

#### Reporting of Thoughts, Feelings, or Behaviors

Clients who record their own thoughts, feelings, and behaviors as part of a CBT-based intervention are able to reflect on their reports and exercise self-monitoring [118]. Self-monitoring is a core feature of many evidence-based psychological therapeutic techniques, including CBT [119,120], mindfulness exercises [121], emotion-focused therapy [122], DBT [123], and acceptance and commitment therapy (ACT) [124]. Self-monitoring can be used to restructure maladaptive anxiety responses [125,126], challenge perpetuating factors of depression [127], and sufficiently treat a small but significant proportion of posttraumatic stress disorder sufferers [128,129].

Self-monitoring is particularly suitable for CBT-based interventions that aim to change behavior, with self-monitoring-only treatment conditions showing benefits for problem drinking [130] and sleep hygiene [131]. Furthermore, self-monitoring is a feature of successful weight loss interventions [132]. Encouraging MHapp users to report their thoughts, feelings, or behaviors in an objective way should therefore help promote accurate, beneficial self-monitoring.

Self-monitoring of mood can boost overall emotional self-awareness (ESA) [133], which can in turn lead to improvements in emotional self-regulation [134]. Emotional self-regulation is valuable for individuals in preventing distress from spiraling out of control and thereby culminating in clinical problems [135]. Poor emotional awareness is a common underlying factor for both anxiety and depression [136]. The ability to differentiate and understand personal emotions, an integral process in ESA, is positively related to adaptive regulation of emotions [137] and positive mental health outcomes [138]. Self-reflection and insight correlate positively with levels of positive affect and the use of cognitive reappraisal, and negatively with levels of negative affect and the use of expressive suppression [139]. Explicit emotion labeling shares neurocognitive mechanisms with implicit emotion regulation ability, suggesting that increasing ESA through practicing labeling of personal emotions will lead to improvements in emotional regulation and adaptation [140].

Some self-monitoring interventions are limited by problems related to recall biases. Self-reflection at the end of a day or in a time and place removed from normal stressors can be inaccurate [141]. One of the benefits of MHapps is that smartphones are capable of ecological momentary assessment (EMA) and experience sampling methods (ESM), which involve measuring experiences and behavior in real time [142]. MHapp users can record self-monitoring data on their smartphones while they are participating in their usual daily routines, undergoing challenges, or directly experiencing stressors [143]. This can help reduce bias in self-monitoring [141], thereby improving the accuracy of users’ reflections.

Increasing ESA should lead to greater help seeking, because factors preventing help seeking include low emotional competence [144] and low self-awareness [77]. Using technology for self-monitoring can increase help seeking, particularly if there is a capacity to contact health professionals built in to the service [145] (see the “Links to Crisis Support Services” section).

Self-monitoring via traditional means might also be less effective for very busy individuals who do not have the time to complete monitoring entries [118]. MHapps can reduce monitoring demands by automating some parts of the monitoring process, such as shifting the burden of some of the more administrative parts of self-monitoring (eg, entering dates and times, formatting monitoring entries) from the user to the smartphone [5]. Using smartphone apps also allows for more frequent and broader opportunities for recording reflections, such as while waiting or traveling on public transport.

Keeping all self-reports structured and objective can help users report quickly and in a format that facilitates data analysis by the MHapp. It may also reduce some of the barriers to self-monitoring: for instance, some depressed clients may find the demands of open-ended self-monitoring overwhelming, whereas perfectionistic or obsessive clients may spend too much time and effort on their monitoring [146]. MHapps with highly structured reporting in a simple interface (see “Simple and Intuitive Interface and Interactions” section) may be able to remedy this by limiting the amount of information necessary for logs, simplifying the monitoring process, reducing the demands on users, and increasing engagement in the app [5].

Several studies support the efficacy of using app-based interventions to increase ESA. Morris et al [147] developed an app that prompted users to report their moods several times a day. Users reported increases in their ESA, and upon reflection of their ratings, some were able to recognize patterns of dysfunction and interrupt these patterns through modification of routines. Kauer et al [133] used a mobile phone self-monitoring program to prompt users to report their emotional state several times throughout the day. Participants who reported on their emotional state showed increased ESA and decreased depressive symptoms compared with controls. Both of these monitoring systems were, however, quite simple and offered little constructive feedback to users about their mood history. They were also trialed on small samples of individuals who had reported psychological distress. There is, therefore, a need to further investigate the impact of smartphone-based mood reporting on ESA and associated mental health outcomes, using an app that gives better feedback and is relevant to nonclinical users.

**Figure 3:**
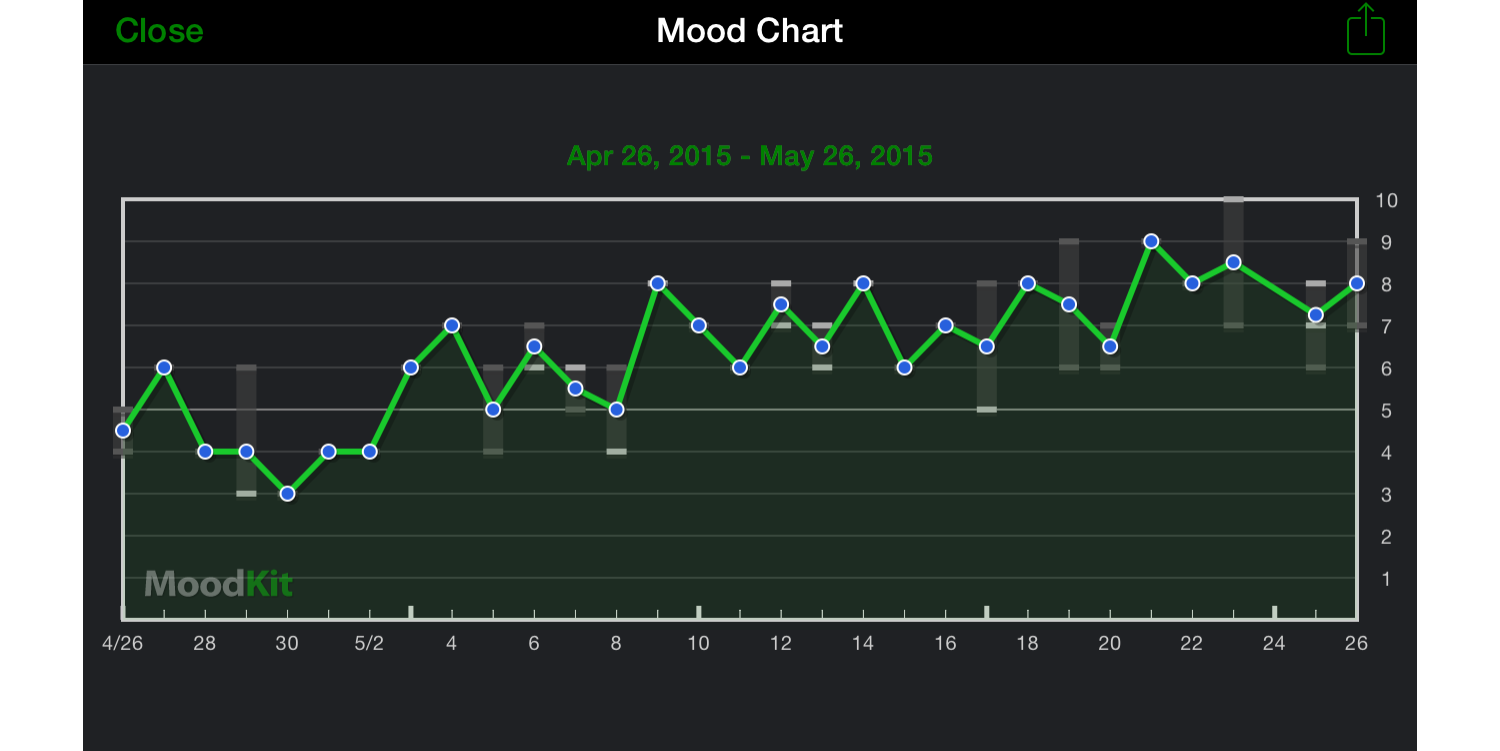
Screenshot of MoodKit displaying mood chart.

The reporting required for self-monitoring can also enable feedback and evaluation of therapeutic progress. In psychological therapy, therapeutic outcomes can be enhanced by providing clients and clinicians with feedback concerning treatment progress [148,149]. These positive effects have been substantiated via a literature review [150] and a meta-analysis, which found a notable effect size (*d*=0.10, 95% CI 0.01-0.19) [151]. MHapps may be able to provide feedback by presenting a user’s own reporting data back to them, but reframed in context with the user’s treatment goal. For example, the mood feedback provided by MoodKit [62] can displayed as a chart, as shown in Figure 3. This type of feedback-focused progress tracking relates also to gamification (see the “Gamification and Intrinsic Motivation to Engage” section) and keeping a log of past app engagement (see the “Log of Past App Use” section).

#### Recommend Activities

CBT aims to engage clients in a range of activities that are congruent with its core principles (ie, context engagement, attention change, and cognitive change) [58]. This represents a shift away from passive interventions toward ones that actively engage clients. CBT-based activities that can be recommended to MHapp users can be summarized into the following categories: (1) exercise and direct mood improvement, (2) behavioral activation, and (3) coping skills training.

##### Activities That Directly Enhance Mood Improvement

A range of activities might target mood directly. For example, it is well established that increasing physical activity and promoting exercise can reduce depressive symptoms [152-154] and anxiety [155], and improve psychological well-being [156,157]. A meta-analysis of 39 RCTs examined the effects of exercise on people diagnosed with a mental illness, and found large effect sizes for depressive symptoms (standardized mean difference=0.80, 95% CI 0.47-1.13) and schizophrenia symptoms (standardized mean difference=1.0, 95% CI 0.37-1.64), and a moderate effect size for quality of life (standardized mean difference=0.64, 95% CI 0.35-0.92) [158]. Effective smartphone apps that promote physical exercise have already been developed [7], but lack an explicit link to mental health that mental-health-focused users may need to justify their use. Motivating MHapp users to engage in physical exercise can have a broad range of mental health benefits.

Another activity that has been directly linked to mood improvement is music listening. Music can be a powerful tool for evoking emotion [159]. Furthermore, relaxing music can challenge emotional recall biases [160] and decrease anxiety [161]. Over 68% of users listen to music on their smartphones [1], and many users use music to reach specific emotional goals [162,163]. An MHapp that includes music listening activities could help users with emotional regulation.

##### Behavioral Activation

Behavioral activation (BA) is a key CBT technique that involves encouraging individuals to engage in physiologically activating and psychologically rewarding activities [164]. A meta-analysis of 17 RCTs reported that BA for clinical depression outperformed control conditions (standardized mean difference=−0.70, 95% CI −1.00 to −0.39) and was as effective as CBT-as-usual (standardized mean difference=0.08, 95% CI −0.14 to 0.30) [165]. There is also evidence that BA can help relieve anxiety [166]. BA aims to (1) encourage the planning of activities and the setting of goals so that clients move away from relying on mood-dependent behaviors; (2) break cycles of avoidance behavior; and (3) develop skills that focus attention on the present moment to enable engagement in activities and associated experiences of pleasure [167]. Motivating MHapp users to complete BA activities is therefore a simple and effective way to improve mental health and well-being outcomes.

Inactivity perpetuates itself via a vicious cycle of low mood: inactivity can lead to decreased opportunities to experience pleasure or gain a sense of mastery, which in turn leads to an increase in negative thinking. This leads to decreased mood, which again leads to greater inactivity, and so forth [168]. BA helps to break this cycle by scheduling activities and reducing escape and avoidance behaviors [167]. Selecting activities that involve mastery and promote positive feelings of self-worth is recommended [168], as such activities can boost motivation via factors related to SDT as well as self-efficacy [100]. Classifying activities as routine, pleasurable, or necessary can be useful, as each has different motivations and benefits to performing [169]. To maximize the likelihood that a recommended behavior will actually be performed by a smartphone user, the behavioral economics of the situation need to be considered [5].

Using a framework such as Fogg’s [170] behavior model, which has been specifically designed with app users in mind, can help in the selection of short, tangible, and universal activities that will maximize user engagement. Fogg’s behavior model states that three factors determine the likelihood of a target behavior occurring: behavior triggers, elements of motivation, and elements of simplicity. Most relevant to selecting BA activities are elements of simplicity, which affect a user’s ability to easily perform the behavior, and include factors such as time, money, physical effort, mental effort, social deviance, and routine. Feedback and self-reflection (see the “Reporting of Thoughts, Feelings, or Behaviors” section) can be an important part of behavioral activation [169]. An app that promotes reflective learning by encouraging an activity and then prompting reflection on the experience immediately after can promote self-discovery [171].

##### Coping Skills Training

Coping skills training is the most direct way of improving self-efficacy [172,173]. Coping self-efficacy (CSE) is a type of self-efficacy reflecting an individual’s perceived ability to effectively cope with adversity and distress [174]. Individuals with high CSE have confidence in their ability to cope with adversity [175] and engage in more active coping strategies [176]. Having greater CSE is associated with better mental health outcomes, including lower likelihoods of depression [177] and anxiety [174], lower overall psychological distress [178-180], and greater psychological thriving [181]. Furthermore, CSE can decrease the negative effect of stressful events on physical health [182]. The greater an individual’s CSE, the less likely they will also be to avoid anxiety-provoking situations [174]. Avoidance plays a key role in the development of anxiety, depression, and many other psychological disorders [183], so interventions that boost CSE by encouraging participation in psychologically beneficial activities will both reduce day-to-day distress and help prevent disorders from developing.

The development of coping skills is a central component in CBT-based practices, and such skills can help clients reduce distress that can trigger problematic maintenance cycles [54,100,104,184]. For example, a core exercise in the treatment of anxiety is the development of relaxation skills, and a meta-analysis of 27 RCTs found a medium to large effect size for relaxation training on anxiety (*d*=0.57, 95% CI 0.52-0.68) [117]. Relaxation training not only develops skills to reduce physiological arousal, but also builds self-efficacy and confidence in coping ability [185,186]. CBT for depression also involves exploration of activities that can reduce distress and improve self-efficacy [187,188]. Research in positive psychology stresses that development of a coping skills repertoire is not only beneficial for those vulnerable to anxiety or depression, but also important for individuals to function well emotionally and achieve their full potential [189]. Offering a range of different strategies and thereby allowing a client to choose which one fits them best can boost self-efficacy and perceived control [190,191]. Furthermore, according to SDT, this choice and control can feed intrinsic motivation toward self-improvement [70].

Unfortunately, there is currently a lack of technology-based interventions designed to develop CSE in relation to mental health. A comparison of 2 Web-based interventions for diabetes management, one involving coping skills training and the other focusing on education, showed that although both interventions had a positive effect on diabetes self-efficacy, only the coping skills (ie, active) intervention showed significant increases in primary control coping behaviors and decreases in perceived stress [192]. Other studies have found no advantage of coping skills training over educational interventions [193-195], but none has investigated the impact of the type of real-time engagement that smartphone apps offer. Many of the coping skills interventions investigated are limited to a series of educational sessions about potential coping strategies. By contrast, smartphone approaches to coping skills interventions could motivate participants to try a number of different coping strategies in real-time as they go about their lives and respond to stressors. This high level of engagement and interactivity could yield substantial improvements in CSE and psychological well-being.

#### Mental Health Information

Psychoeducation, an integral part of CBT, presents clients with mental health information in an attempt to teach them about the psychological processes underlying their distress and inform them of resources available to manage it [196]. A meta-analysis of 25 RCTs reported that the “Coping with Depression” psychoeducational intervention, developed by Lewinsohn et al [197], was effective at treating depression, albeit with a small effect size (*d*=0.28, 95% CI 0.18-0.38) [102]. Participants who completed the preventative version of the intervention were 38% less likely to develop clinical depression [102]. Psychoeducation can also improve mental health outcomes on a community-wide scale. A meta-analysis of 15 studies concluded that the Mental Health First Aid program, developed by Kitchener and Jorm [198], improved participants’ knowledge (Glass’s Δ=0.56, 95% CI 0.38-0.74), attitudes (Glass’s Δ=0.28, 95% CI 0.22-0.35), and supportive behaviors (Glass’s Δ=0.25, 95% CI 0.12-0.38) with regard to mental health [199].

MHapps are well positioned to deliver psychoeducation, as they can engage users with a range of multimedia and audiovisual tools to aid understanding of mental health concepts. A meta-analysis of 4 RCTs reported a small effect size (*d*=0.20, 95% CI 0.01-0.40) for passive psychoeducation including brief audiovisual sources and information presented via the Internet, demonstrating that even this minimal form of psychoeducation is effective at reducing depressive symptoms and psychological distress [200]. Another meta-analysis of 19 studies found a significant but small effect size of psychoeducation on stress (standardized mean difference=0.27, 95% CI 0.14-0.40); in a follow-up moderator analysis, this study showed that shorter interventions were significantly more effective than were longer interventions (*P*<.05, B=−0.020, 95% CI −0.024 to −0.016) [201]. Smartphones are well equipped to deliver this kind of brief, passive psychoeducation, and MHapps can offer links to websites for more in-depth information where required [202].

Psychoeducation topics that have greater relevance to the user’s reported problems are of greater use to the user, so MHapps should tailor psychoeducation to individual users (see the “Automated Tailoring” section) [111]. For example, if a user reports feelings of anxiety, delivery of information about the physiological responses of anxiety and their relationship with thoughts and behaviors would be more appropriate than would delivery of information about the physiological symptoms of depression. Relevance and engagement may also be enhanced by adopting a collaboratively empirical approach [64], whereby users are encouraged to apply concepts learned through psychoeducation to their own circumstances through hypothesis testing. An app that engages users in a process of experimentation-based self-discovery may enhance psychoeducational outcomes.

Presenting mental health information and engaging individuals in psychoeducation can lead to boosts in mental health literacy (MHL) [203]. MHL has been defined as “knowledge and beliefs about mental disorders which aid their recognition, management or prevention” [204]. Greater MHL is associated with a reduction in stigmatizing beliefs about those with mental illness [205] and with greater and more appropriate help seeking [144,206,207]. Known factors preventing young people from seeking help for mental health issues include poor MHL, preference for self-reliance in problem management, and perceived stigma of mental illness [77].

Mental health information can also increase treatment credibility, thereby motivating users to engage with a given treatment [208], and can provide evidence-based justifications for performing recommended activities (see the “Recommend Activities” section). Notably, users have a tendency to perceive health information on the Internet as being credible [209], so this raises the ethical imperative of ensuring that all information is strictly evidence based. Providing links to sources of evidence may satisfy the needs of scientifically minded users and mental health experts. The wealth of mental health resources already available online [210,211] could be utilized by MHapps. Improving MHL may simply be a case of providing easy access to these resources through the app.

Christensen et al [212] compared 2 Web-based interventions aimed at promoting mental health. BluePages, a psychoeducation site, and MoodGYM, a self-guided CBT site, both led to decreases in users’ depression symptoms. MoodGYM reduced users’ dysfunctional thinking, whereas BluePages failed to do this. However, BluePages improved users’ knowledge of treatments for depression beyond what MoodGYM achieved. This evidence suggests that both psychoeducation and self-guided CBT interventions are needed to generate the most substantial and stable gains in mental health and well-being. A successful app-based intervention would combine elements of both psychoeducation and self-guided CBT.

#### Real-Time Engagement

The high engagement potential of smartphones means that users are able to seek help for psychological challenges in the moment they are experiencing them or soon after. MHapps that have not been designed to be used in real time will fail to capitalize on valuable opportunities to engage with users.

Many CBT-based therapy programs utilize in vivo exposure and between-session (homework) activities to help clients resolve maladaptive anxiety responses in ecologically valid settings [65,105]. The advantages of between-session interventions are wide ranging [66] and have already been covered in this paper under Recommendation 1 “Cognitive Behavioral Therapy Based.” Some therapy programs have even utilized virtual reality to harness the power of real-time engagement [213,214]. These interventions acknowledge the benefits of engaging with clients in real-world contexts in real time.

The rationale behind real-time engagement includes basic behavioral principles of learning. It enhances the generalization of learned skills to new settings, and can encourage practice of behaviors to maintain therapeutic gains [215]. Real-time engagement opens up more opportunities for learning and applying coping strategies in ecologically valid contexts. Of the MHapps that aim to increase users’ coping abilities, few utilize the real-time capabilities of smartphones [8,216]. Most deliver long-running interventions designed to increase users’ overall resilience or optimism, such as SuperBetter [59]. The MHapps that do provide users with in vivo coping strategies, such as MindShift, are very clinically focused, which restricts their reach (see the “Designed for Nonclinical, Nondiagnostic Support” section). Engaging users to attempt coping strategies in real time improves the functionality of the MHapp and increases opportunities for learning.

Heron and Smyth [217] call health apps that use real-time engagement “ecological momentary interventions,” and they present evidence for the efficacy of such apps in psychosocial applications. Depp et al [110] developed and trialed a mobile intervention called PRISM that used real-time data to prompt individuals with bipolar disorder to engage in self-management behaviors. The results from this study were promising, but this rather clinically focused intervention was built for PDAs rather than for smartphones, and therefore was unlikely to be as unobtrusive in daily life as smartphone interventions.

#### Activities Explicitly Linked to Specific Reported Mood Problems

Linking recommended activities to specific psychological challenges helps trigger engagement with an intervention. Eyal [3] emphasizes the need for successful apps to have triggers that fulfill an immediate and obvious need, using the metaphor of vitamins and painkillers. Vitamin-like products do not satisfy immediate needs but are espoused as beneficial, whereas painkiller-like products give users immediate benefits. MHapps like SuperBetter [59] and Happify [218] require users to engage with the app regularly and encourage them to do so by reminding them of the benefits offered by the app. However, the activities recommended by these apps are not directly linked to any specific mood problems that users may be experiencing. Using specific problems as triggers can strengthen engagement [3] and can help in the learning of targeted coping strategies.

Utilizing habit formation can be a very effective way of guaranteeing repeated engagement with an app, which in the case of MHapps, should lead to mental health benefits. Habits are repeated behaviors that are triggered by cues [5]. To generate a habit that involves using an MHapp, a cue must be selected to associate with app use through the processes of conditioning [3]. Using mood problems as cues can drive real-time engagement (see the “Real-time Engagement” section). For example, an MHapp that is designed to be used when a user is feeling low or anxious is better suited to habit formation processes than is an MHapp that offers no cues for engagement and expects users to engage with it randomly throughout the day. Habit formation will also be driven if an MHapp is linked to activities that decrease psychological distress, increase self-efficacy, or reward users in some other way [5].

#### Encourage Nontechnology-Based Activities

When designing interventions for smartphones, it may be tempting to build the therapeutic activities into the app’s interface. However, this goes against the ethos of CBT-based practice, which emphasizes the important role of activities and interventions outside of contact with a practitioner, computer program, or self-help guide [120]. Encouraging users to engage in real-world activities, off the device they are using, respects that ethos and fosters the environmentally valid application of skills.

In this context, it is also of note that depression and lower psychological well-being are correlated with Internet use, especially among introverts with low levels of social support [219]. However, this role is moderated by the function of Internet use—for instance, Internet use for communication has been found to be related to lower levels of depression, whereas Internet use for noncommunication purposes has been found to be related to greater depression and social anxiety symptoms [220]. Internet use and Internet addiction have also been associated with social anxiety [221], and positive correlations have been found between avoidance coping and Internet use [222,223]. This may also apply to Internet-enabled, noncommunication-based mobile phone apps that distract users’ attention away from psychological challenges. Avoidance coping has been shown to increase the likelihood of acute and chronic life stressors and depressive symptoms over long periods [224]. Providing users with nontechnology-based activities helps to balance MHapp-based technology use with positive behavior change strategies and limits use of avoidance coping strategies.

Technology can allow greater multimodal learning by combining text with spoken language, sounds, and graphics that are closer representations of learning in an applied setting [225]. For example, blended learning, which involves blending the use of technology with applied learning in the classroom [226,227], has been shown to deliver superior learning outcomes to traditional teaching methods [228,229]. It has been recommended that technology be used to enhance real-life experiences, not replace them [230,231]. MHapps may therefore harness the power of blended, multimodal methods to effectively enhance learning of real-world coping strategies.

Some available MHapps encourage users to engage in nontechnology-based activities. SuperBetter motivates users to engage in regular nontechnology-based resilience-building activities [232]. Preliminary results from an RCT suggest that SuperBetter is effective for reducing symptoms of depression [233]: specifically, SuperBetter users experienced a reduction in the equivalent of 5 symptoms of depression, and waitlist participants experienced a reduction in just 2.

#### Gamification and Intrinsic Motivation to Engage

The therapist plays an instrumental role in promoting clients’ motivation to engage in psychotherapy and undertake homework activities [65]. This means that self-help CBT may be of limited use if the user suffers from low motivation and volition, which is common among those with mood disorders [234]. Gamification is a novel solution that may help counteract problems with motivation and yield additional well-being outcomes.

**Figure 4:**
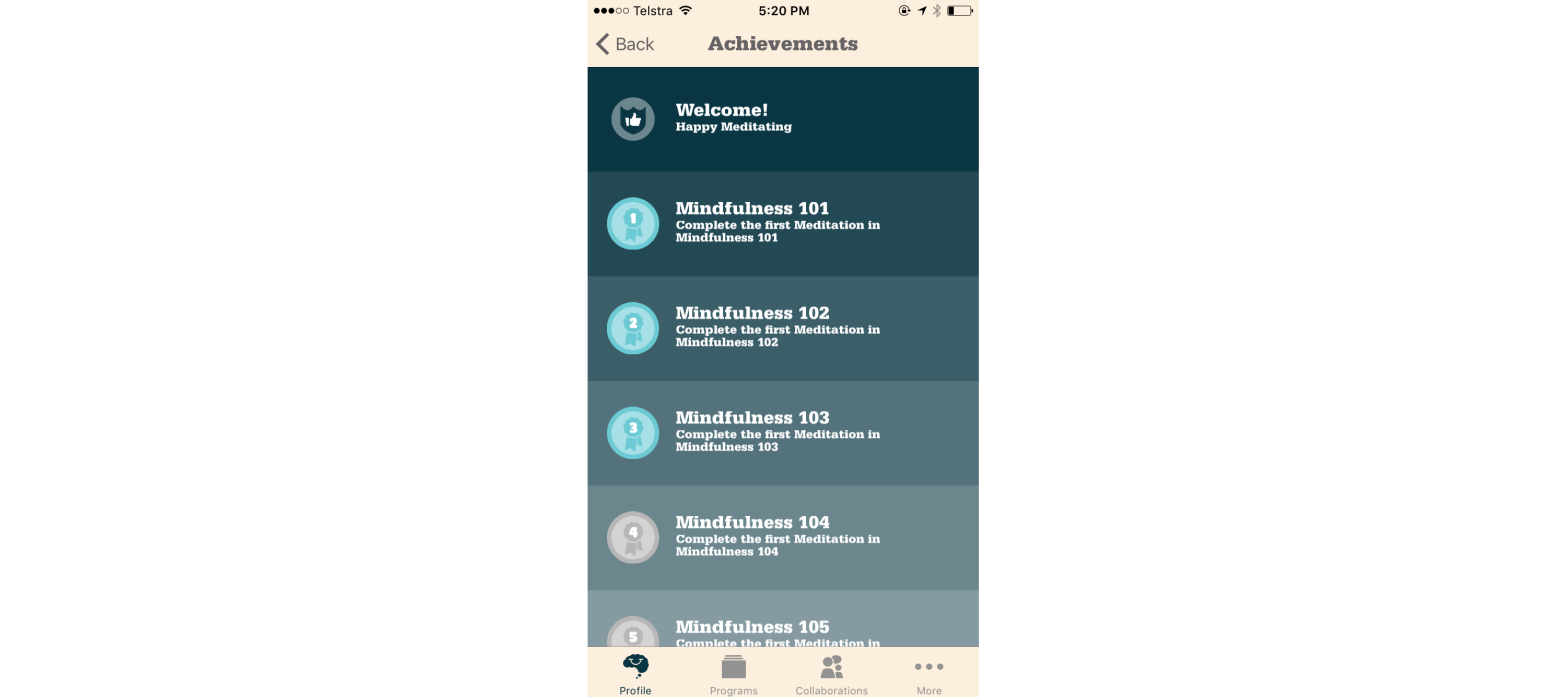
Screenshot of Smiling Mind displaying achievements.

To “gamify” something does not mean to turn it into a digital game. Gamification is instead the use of “game-based mechanics, aesthetics, and game thinking to engage people, motivate action, promote learning, and solve problems” [17]. Many apps have employed the principles of gamification to motivate users to pursue various goals, but such goals are likely to be most motivating if they originate from the users themselves [235]. Gamification can enhance a user’s motivation to pursue an existing goal, but it does not, in itself, create new goals for users. These goals may require the formation of new routines, and gamification excels at motivating people to repeat tasks until new habits are formed [3]. Some examples include Nike+ Running [236] and other fitness tracking apps that award points for reaching fitness goals, and Smiling Mind [60], which tallies minutes spent meditating and awards badges for specific meditation-related achievements, as seen in Figure 4.

Games are abstracted, simplified versions of reality, so gamification can help users reduce reality’s complexity into a more easily understood operating model [17]. This helps users to quickly learn cause-and-effect inferences, without complex extraneous factors detracting from their motivation to make change. Gamification is also based on the principle that making something goal oriented can increase the positive feelings associated with it and drive intrinsic motivation [232]. In this context, gamification is an applied expression of the concepts proposed by SDT [17] (see the “Cognitive Behavioral Therapy Based” section).

Gamification is a means of making intrinsic rewards more obvious and tangible. Alternate reality games (ARGs) link online or app-based events and achievements to real-world ones [237]. By tracking and quantifying the progress of real-world goals, users are able to reflect on their competency and experience mastery. Gamification also helps to break larger, more abstract goals down into smaller, more tangible and concrete tasks. For example, if a user’s goal is to build resilience and recover from depression, the MHapp and ARG SuperBetter is able to break that goal down into daily tasks of activating 3 power-ups, battling 1 bad guy, and doing 3 quests [59]. Although many regular electronic games are attractive because they are escapist [238], ARGs are antiescapist, motivating users to deal with real-world challenges and increasing the likelihood of them obtaining intrinsic rewards.

Individuals tend to choose more challenging activities when these activities are framed as games and imbued with intrinsic motivation [239,240], and making activities goal-directed further enhances enjoyment of their challenges [241]. When building points and award systems for gamified solutions, it is best to introduce users by awarding them some points or rewards on sign-up or early on. The endowed progress effect means that starting with some points rather than zero increases effort and motivation to engage [242].

Although fun is the primary reward in electronic games, self-efficacy is the primary reward in well-structured gamified solutions [235]. Gamification principles can amplify achievements by offering immediate reflections of intrinsic rewards, thereby boosting self-efficacy. Badges, points, and other gamification rewards remind users that they have achieved something by quantifying their success and allowing users to reflect on their own growth [232]. Even apparent failure can be rewarding in a gamified environment, if the right animation or interaction—namely, one that maintains the user’s feelings of competency—is used [17].

One study found that the reward- and motivation-related neurotransmitter dopamine was released during a simple, goal-directed game-based task, presenting neurological evidence for why game-based mechanics may yield positive well-being effects [243]. A meta-analysis of 10 RCTs found that electronic-game-based depression interventions had a moderate effect on depressive symptoms (*d*=−0.47, 95% CI −0.69 to −0.24) [244].

Apps allow constant improvement through updates and Web-delivered content [245], and this is very important for a successful gamified solution. Not only should the gamified structures be tweaked until users are being optimally engaged, but also novel and untried features should be introduced to motivate users to maintain their engagement with the app. Apps that sustain variability throughout use can maintain user interest with the promise of new and interesting content [3].

#### Log of Past App Use

Gamification relies on users having the ability to record and review their achievements. Thus, having a well-presented log of past app use can potentially raise intrinsic motivation and increase users’ investment in the app. Logs of past use can also enable automated tailoring (see the “Automated Tailoring” section). If a log is being recorded for this purpose, then making it accessible to the user should not present coding difficulties.

Narratives in games can link discrete, seemingly unrelated tasks [232]. Narrative framework embedded into an app’s use can motivate users to do small tasks to work toward an overall goal. Using a log that provides users with useful feedback about their successes and challenges can provide this narrative framework. For example, many mental health boosting activities, such as exercise, relaxation, and cognitive reframing, appear to be unrelated. However, embedding them into a narrative that has an end goal related to boosting mental health can help users make sense of the tasks, thereby boosting users’ motivation to achieve these goals.

Wilson’s [246] story-editing technique can be applied to apps to enhance engagement [5]. According to Wilson’s theory, reinterpretation of a self-narrative can affect future behavior. Past failings can be reinterpreted as learning opportunities, and other actions can be framed as preparations for a specific goal. Altering self-narrative in this way helps users see “themselves as someone for whom the action is a natural, normal extension of who they are” [5]. For example, fitness trackers and apps that count a user’s steps, such as the Jawbone UP [247], show users that they have already been exercising, but may need to increase their level slightly to achieve their goals.

The addition of more storyline-based game principles, such as avatars with experience points, can further reinforce a sense of narrative [17]. Avatars are characters within a game that are representations of the user [248]. Bandura’s [249] social cognitive theory states that the relatability and similarity of a model will increase the likelihood of a learned behavior being performed. Fox and Bailenson [250] substantiated this in a digital environment, with participants exercising more when they were shown an exercising avatar that resembled them than when the avatar did not resemble them. Furthermore, users who are given taller avatars act more confidently and aggressively than do those who are given shorter avatars, both virtually and face-to-face [251,252]. This indicates that the narrative elements used in a gamified solution can translate to behavioral changes in the real world. If users are capable of exercising autonomy and customizing their avatars so that these avatars better resemble users' ideal state, the likelihood of behavioral modification should be improved.

Importantly, users must also be aware of the cognitive or behavioral work they have completed. Investment through labor and work increases engagement and enjoyment [253]. Understood through SDT, this may be a reflection of a user’s desire to build competency and mastery [254]. Therefore, users who can log the extent of their app use and receive feedback on how much they have done or invested are more likely to have greater, more enjoyable engagement with the app.

To maintain a log of app-based activity, users may have to create an account to synchronize their app progress with a server. This would allow users to use multiple devices and help them avoid losing their progress if their app were deleted or they changed devices. Many apps use a social networking site login, such as Facebook, for easy account creation, but this can trigger privacy-related anxieties in users [255], so it may be best to avoid this when creating an MHapp that collects potentially sensitive data. Other ethical and privacy concerns arise when recording app data to a server [256], so the integrity of storage sites should be thoroughly evaluated, especially with regard to obtaining users’ informed permission to record and access their personal data [116].

#### Reminders to Engage

Some of the most successful guided self-help Web-based treatments for anxiety and depression use email or telephone reminders to maintain user engagement [10]. Reminders can increase adherence and reduce dropout from self-help CBT interventions [24]. Push notifications are alerts that can be sent via the Internet to apps on mobile devices [257]. MHapps that use push notifications are similar to Internet interventions that use short message service (SMS) reminders in that they prompt users throughout their day to engage in the intervention. Previous studies have demonstrated that interventions with SMS reminders can be effective for diabetes management [258], smoking cessation [259], and weight loss [260].

Although external triggers can be useful to remind users of an app, too many annoying or interruptive reminders can lead to disengagement. SDT stipulates that anything that quashes a sense of autonomy, such as a series of insistent reminders, can reduce intrinsic motivation to engage [71]. Eyal [3] distinguishes internal and external triggers of engagement, extoling the long-term benefits of the former over the latter. External triggers may help to initiate the engagement processes, but internal triggers are more reliable drivers of long-term habits. Eyal cites the example of social image-sharing app Instagram, which uses the internal trigger “I want to share this experience with others.” However, if Instagram reminded users every day to post an image, it is likely that using it would soon be perceived as a chore with no intrinsic reward.

Although some reminders can restrict a sense of autonomy, others can encourage it. A recent meta-analysis of 42 studies found that phrases that emphasize an individual’s right to refuse, such as “But you are free to accept or refuse,” increase the likelihood of people agreeing to requests, with an overall effect size of *r*=0.13 [261]. External reminders should be framed within an SDT context to grant autonomy and respect intrinsic motivators. Chaiken’s [262] heuristic-systematic processing theory can further inform the design of reminder communications. Framing reminders to satisfy the commitment and consistency, liking, authority, or scarcity heuristics can aid user engagement [263].

#### Simple and Intuitive Interface and Interactions

The simplicity of a program’s interface and ease of navigation significantly influence user perceptions of quality in Web-based mental health interventions [264,265]. User satisfaction and perceptions of credibility directly influence engagement and therapeutic benefit [208]. Building an enjoyable app with good graphic design and a slick, intuitive, and satisfying interface is necessary for an effective intervention [5,266]. Simplicity also reduces the likelihood of technical difficulties that may dissuade users from engaging [267].

Fogg’s behavior model (ie, the model of technology-based behavior change [268] discussed in the “Recommend Activities” section) emphasizes that simplicity reduces demands for initiating behavior outcomes, and increases the likelihood of a behavior occurring. A simpler interface decreases the ability required to engage with the app, and increases the likelihood of successful engagement [3].

No-action default (or “opt out”) options have enormous influence over the use of a product or service [269]. For example, countries that have presumed consent organ donation policies have 25-30% higher donation rates after all other factors that influence rates are accounted for [270]. It has been argued that making organ donation as the no-action default option for Australian citizens could significantly raise donation rates and save many lives [271]. No-action defaults both preserve autonomous decision-making and influence behavior toward goals [272], so MHapps are well positioned to capitalize on these effects to guide users toward beneficial outcomes. App settings should be customizable to allow for autonomous use and tailoring, but come with recommended default options preset. For example, the default option for reminders should be set to “on,” and at a frequency that is not overwhelming for the user (see the “Reminders to Engage and External Triggers” section).

The language used in the delivery of a mental health intervention, particularly a self-help intervention, can also have a major impact on engagement [273]. The language needs to be simple, concrete, confident, and hopeful for users to understand and engage with interventions. Language should also be inclusive of all sexual orientations and lifestyles [274] and be nonclinical, nonpsychopathological, and nondiagnostic to avoid stigma [57,99]. The literacy of intended users must be considered, just as it is for different newspapers [275]. The length of sentences and paragraphs is not only limited by the constraints of a smartphone screen, but also by the working memory of users. Making information meaningful to users can help its consolidation into memorable chunks, easing the demands on memory [276]. Using illustrations, such as faces, for emotions, can also improve the efficiency of understanding [277]. Decreasing load on memory is all the more important for users suffering from symptoms of depression or anxiety, which can restrict working memory function [278].

Although keeping information simple is necessary for initial understanding, enabling exploration of more in-depth information is important to satisfy some users [202]. Building a feature such as a “learn more” or “help” button into an MHapp can enable users to access more information about certain content or features. Furthermore, navigation around an app can be key to maintaining a sense of autonomy and competency. An app that limits a user’s freedom of navigation may be frustrating and not intrinsically rewarding to use. Features such as an ever-present button that navigates the user back to the home screen can remedy this.

#### Links to Crisis Support Services

Crisis support services are valuable resources for vulnerable individuals undergoing acute psychological distress [279]. Suicidal callers to crisis hotlines experience significant decreases in suicidality, hopelessness, and psychological pain [280]. Developing and utilizing these services has consequently become a key area for promoting public mental health care [281,282]. However, barriers to help seeking can prevent troubled individuals from utilizing these supports.

Building links to crisis support services into MHapps may overcome some of these barriers. Furthermore, an MHapp that records a user’s mood (see the “Reporting of Thoughts, Feelings, or Behaviors” section) may be able to unobtrusively detect indicators of depressive episodes and prompt contact of the relevant supports. Negative attitudes toward seeking help can be a major barrier to engagement [77]. However, if an app presents support options in an attractive and easy-to-access way, accessing those supports is more likely to be perceived as acceptable and appealing [269]. Lack of awareness of service availability, or the nature of support offered, can also prevent help seeking [203], as can the belief that support is rarely available and will not help anyway [283]. An MHapp that enables access to information about how support services operate and how they can help could reduce these barriers. According to the Fogg’s behavior model [268], accessing crisis support services through technology should be made straightforward to reduce barriers to action and increase the likelihood of service contact being made.

Importantly, Internet supports are preferred to telephone helplines in some populations, including young people [284]. Organizations such as Lifeline have an online crisis support chat facility [285], so where these are available, links should be offered on mobile devices. There is also growing support for the effectiveness of online chat options [286], which may be better suited to how some individuals who use digital devices tend to communicate [287].

#### Experimental Trials to Establish Efficacy

A major shortcoming of currently available MHapps is the lack of RCT evidence for their efficacy. Although many apps use evidence-based frameworks, like CBT, only a handful have been experimentally trialed. Donker et al [8] conducted a systematic review of the literature, searching for evidence of effective MHapps; only 8 papers were identified as providing scientific support for MHapps, and in these papers, only 5 separate MHapps were described. Just 1 of these 5 was a self-contained app, with the other 4 requiring input from a mental health professional. Frustratingly for those who might benefit from these apps, none of them is currently available on the iOS or Android app stores.

This lack of controlled outcome research in the field is unexpected, given the ease of collecting data using mobile and Internet technologies [90]. Although validation of other psychological interventions requires time-consuming assessments, MHapps are capable of reliably, quickly, and automatically collecting a myriad of self-report and behavioral usage data [288].

When starting with a product vision for an app, target outcomes should be well defined in concrete, objective, and measurable terms [5]. These overarching goals guide development and enable a definition of success for the app. There are three main types of data that can be used to assess the target outcomes of MHapps: (1) assessment tools administered before and after a set period of app use, (2) EMA techniques to administer multiple brief self-report questionnaires throughout app use, and (3) app usage data. A thorough assessment of an MHapp should attempt to use all three data sources.

##### Assessment Tools Administered Before and After a Set Period of App Use

Wendel [5] stresses that, where possible, target outcomes for apps should avoid user “states of mind,” such as emotions and other internal, psychological variables, as these are problematic to measure. However, the main goal of MHapps is to alter the user’s state of mind. This means the tools used to measure the MHapp’s target outcomes should be selected carefully, keeping in mind the ease of administration via a smartphone, the ease of integration into an MHapp’s interface, the licensing of the assessment tool, and the validity and reliability of the measure.

Outcome measures for MHapps should contain a suitable assessment of emotional well-being and mental health. For example, the 9-item Patient Health Questionnaire (PHQ-9) [289] is a brief, self-administered, valid, and reliable measure with 88% specificity and 88% sensitivity for major depression. It is licensed to be used freely, and existing apps have successfully adapted it for a smartphone interface [290]. The 7-item Generalized Anxiety Disorder scale (GAD-7) [291] is a similar measure for anxiety, and using both the PHQ-9 and GAD-7 together can give a balanced assessment of emotional psychopathology [292]. To assess the languishing-flourishing dimension of mental health, the 14-Likert-item Warwick-Edinburgh Mental Well-Being Scale could be used, as it is a brief, reliable, and valid tool [293].

Secondary to mental health outcome measures are measures of the MHapp’s intervention targets. For example, a self-monitoring MHapp should aim to assess the degree to which insight and ESA are being enhanced by the self-monitoring intervention (see the “Reporting of Thoughts, Feelings, or Behaviors” section). To validate their MHapp, Kauer et al [133] used a short survey, delivered by phone, called the ESA Scale. This tool comprises 33 items, all rated on a scale from 0 (never) to 4 (a lot), and was adapted from the 20-item Self-Reflection and Insight Scale [294], the 10-item Ruminative Response Scale [295], and the 12-item Meta-Evaluation Scale [296]. MHapps that aim to boost CSE (see the “Recommend Activities” section) could use the Coping Self-Efficacy Scale [175], which is a short questionnaire that can be administered via a smartphone. MHapps that utilize elements of psychoeducation may require assessments of MHL (see the “Mental Health Information” section). There is no standardized assessment tool for MHL, but it is often measured using self-report questionnaires and vignettes [204], which can be adapted for smartphone-based assessment. However, vignettes tend to be long and cumbersome forms of assessment, and are not well-suited to the restrictions of smartphone screens and interfaces. A well-validated, standardized, brief assessment tool for MHL would benefit the development of many self-help interventions, including MHapps.

It is recommended that follow-up data are collected at several different time points throughout the MHapp intervention and after its use has been concluded. An RCT on the mindfulness meditation app Headspace [297] found that it led to increases in positive affect and decreases in depression, but had no effects for measures of negative affect, satisfaction with life, or flourishing. This failure to uncover effects may be attributable to the limited time course of the research, as the intervention only lasted for 10 days and there was only one postintervention measurement [298].

##### Ecological Momentary Assessment

Using EMA, brief self-report questionnaires can be prompted at various periods throughout a user’s day [143], with the precise time of survey completion accurately recorded. EMA can reduce bias in self-report data [142] and enables study of ecologically valid contexts [141]. As described in the “Reporting of Thoughts, Feelings, or Behaviors” section, EMA can also be a valuable part of interventions.

It is important to adopt an EMA design that is most appropriate for the types of data being collected and for the MHapp being trialed. EMA questionnaires should be brief enough for smartphone users to feel capable of completing them without too much interruption to their day. The aim of EMA is to obtain an ecologically valid measurement, so limiting disruption maximizes validity [217]. The design of EMAs can be event-based or time-based, depending on whether responses are collected following a specific event, such as an app-based interaction, or triggered at a given time point [141]. The choice in design should also be well thought-out and justified. For example, if a time-based EMA collects measurements at the exact same time every day, it may not accurately capture changes in the user’s state experienced throughout the rest of the day. Event-based EMA should be used in an MHapp that recommends activities (see the “Recommend Activities” section) and requests a user to rate their mood before and after performance of the activity (see the “Reporting of Thoughts, Feelings, or Behaviors” section).

##### App Usage Data

Ongoing monitoring of client data is valuable to the validation of CBT-based interventions [142], and ongoing data collection should be a seamless and constant background process on smartphone apps. App usage data are often collected continuously by app developers to analyze user behavior and improve app functionality. The range of data capable of being collected in this way is very large, including measurements such as time spent using specific features of an app, number of times the app is used in day, and what times in the day features on the app are being used.

Data collected via EMA and other assessment tools may also provide insight into user variables that affect patterns of app usage. For example, it may be found that a specific feature is used most when users are highly distressed. This is an important information to consider, for both the development of psychological theories and the development of MHapps, as it may be appropriate to display a link to crisis services on the app’s interface when a specific feature is being used.

Program adherence is easily assessed with usage data, and app design can be concurrently altered to increase adherence [24]. Although there is no doubt that these data are already being used by developers to improve individual MHapps, there has seemingly been a lack of academic transparency to validate those MHapps and aid in the development of others.

### Strength of Evidence for Recommendations

Each recommendation explored in this review is supported by a different rank of evidence. Table 2 summarizes the 16 recommendations and ranks each according to evidence strength. The strongest level listed includes recommendations that are demonstrably effective, as shown by the numerous meta-analyses and RCTs of interventions previously cited in this review. However, more research in the form of RCTs is needed for such MHapps. The next rank of evidence pertains to recommendations that are probably effective according to available evidence but still require more research in the MHapp field. The rank under this includes recommendations that appear to be promising according to the evidence, but, again, must be researched in more depth to validate their stated principles in self-help interventions, including MHapps.

## Discussion

MHapps offer exciting new opportunities to improve and manage the mental health of smartphone users. This review has generated 16 recommendations to be considered in the development of future MHapps. In summary, MHapps should aim to prevent emotional mental health problems by employing a wide array of CBT-based techniques that are tailored to an individual’s needs and delivered via a simple, interactive design. Structures of gamification and habit formation should be used to maximize engagement in the app’s interventions. The app itself should be experimentally validated, and user data should be utilized for its ongoing improvement.

It is highly recommended that MHapp developers familiarize themselves with the literature, both in the field of self-help CBT and in the field of app-based behavior change, before embarking on any MHapp projects. Respecting the value of both of these research fields should enable the reliable, engaging delivery of an evidence-based mental health intervention. This review may help developers get started with this familiarization process, but further reading is strongly advised. Furthermore, a multidisciplinary team consisting of experts in app usability engineering, programming, data collection and analysis, industry and health care sector applications, clinical psychological interventions, and any other relevant fields is strongly advisable.

The Mobile Application Rating Scale (MARS) is a recently developed measure enabling objective, multidimensional rating and comparison of mobile health apps [299]. Tools such as this will be essential for the future of MHapp development, and will enable clinicians and consumers to make more informed decisions about their choice of smartphone-based support.

There is a risk of researchers developing MHapps primarily for research needs rather than to meet the needs of end users. When an MHapp is released to the public, it is a self-contained product and must operate efficiently in the user’s daily routine. For MHapp research to be ecologically valid, MHapp developers must create self-contained apps that still function outside of a research setting. Several RCTs have been conducted on MHapps that are not publically available [52]. This prevents researchers and intervention developers from analyzing and exploring existing evidence-based MHapps. It also blocks help seekers from finding evidence-based MHapps and benefiting from effective support.

**Table 2:** Recommendations for future mental health apps.

Evidence	Recommendation	Details
Demonstrably effective, but more research needed in MHapp field	1. Cognitive behavioral therapy based	Start with an evidence-based framework to maximize effectiveness
	2. Address both anxiety and low mood	Increases accessibility and addresses comorbidity between anxiety and depression. Also compatible with transdiagnostic theories of anxiety and depression
Probably effective, but more research needed in MHapp field	3. Designed for use by nonclinical populations	Avoiding diagnostic labels reduces stigma, increases accessibility, and enables preventative use
	4. Automated tailoring	Tailored interventions are more efficacious than is rigid self-help
	5. Reporting of thoughts, feelings, or behaviors	Self-monitoring and self-reflection to promote psychological growth and enable progress evaluation
	6. Recommend activities	Behavioral activation to boost self-efficacy and repertoire of coping skills
	7. Mental health information	Develop mental health literacy
	8. Real-time engagement	Allows users to use in moments in which they are experiencing distress for optimum benefits of coping behaviors and relaxation techniques
Supported by theory and indirect evidence but focused research needed	9. Activities explicitly linked to specific reported mood problems	Enhances understanding of cause-and-effect relationship between actions and emotions
	10. Encourage nontechnology-based activities	Helps to avoid potential problems with attention, increase opportunities for mindfulness, and limit time spent on devices
	11. Gamification and intrinsic motivation to engage	Encourage use of the app via rewards and internal triggers, and positive reinforcement and behavioral conditioning. Also links with flourishing
	12. Log of past app use	Encourage use of the app through personal investment. Internal triggers for repeated engagement
	13. Reminders to engage	External triggers for engagement
	14. Simple and intuitive interface and interactions	Reduce confusion and disengagement in users
	15. Links to crisis support services	Helps users who are in crisis to seek help
Necessary for validation of principles	16. Experimental trials to establish efficacy	It is important to establish the app’s own efficacy before recommending it as an effective intervention

A behavioral plan is a “detailed ‘story’ of how the user progresses from being a neophyte to accomplishing the action while using the product” [5]. Any app should be designed from the foundation of a comprehensive behavioral plan [5]. This means that it may not be possible to incorporate all 16 recommendations listed herein into a single MHapp. To guide development of behavioral plans and interactive frameworks, it would be helpful to focus on specific foundations. Three of the recommendations listed can be used as foundations for intervention development, as they aim to target specific psychological constructs, such as ESA, MHL, and CSE. The “Reporting of Thoughts, Feelings, or Behaviors” section details mood reporting, self-monitoring, and improving ESA. MHapps that use this as a foundation could be referred to as “reflection-focused.” The “Recommend Activities” section relates to engaging users in activities to improve their CSE. MHapps that use this as a foundation could be referred to as “goal-focused.” The “Mental Health Information” section relates to mental health information, psychoeducation, and improving MHL. MHapps that use this as a foundation could be referred to as “education-focused.” More research is needed to investigate the different effects of reflection-focused, education-focused, and goal-focused MHapp designs on mental health, and whether different users obtain different benefits from each design.

Each recommendation explored in this review could be the target of an RCT. RCTs that compare identical MHapps with or without specific features could provide evidence for or against these features in future MHapps. However, it is important to acknowledge the influence of the overall behavioral plan on the MHapp’s effectiveness. Some features may work better in one MHapp’s behavioral plan than in another’s, and simply including more recommended features may not improve the overall intervention. Future MHapp and eHealth RCTs should aim to validate underlying theories and principles for intervention improvement [21].

The World Health Organization [300] predicts that depression will become the global leading cause of disease burden by 2030. There is an enormous worldwide need for better preventative mental health, and MHapps that target emotional well-being are set to provide exciting new opportunities in the field. The evidence-based recommendations discussed herein are important for all MHapp developers to acknowledge if better interventions are to be developed to meet this rising demand in the future.

## Abbreviations

ACT: acceptance and commitment therapy
ARG: alternate reality game
BA: behavioral activation
CBT: cognitive behavioral therapy
CCBT: computerized cognitive behavioral therapy
CE: collaborative empiricism
CSE: coping self-efficacy
DBT: dialectical behavior therapy
EMA: ecological momentary assessment
ESA: emotional self-awareness
ESM: experience sampling method
GAD-7: 7-item Generalized Anxiety Disorder Scale
MARS: Mobile App Rating Scale
MHapp: mental health app
MHL: mental health literacy
PDA: personal digital assistant
PHQ-9: 9-item Patient Health Questionnaire
RCT: randomized controlled trial
SDT: self-determination theory
TCBT: transdiagnostic cognitive behavioral therapy
UP: unified protocol
